# Supplementary figures and images for: Estimating the undetected emergence of COVID-19 in the US
Source: PLoS One. 2023 Apr 6;18(4):e0284025. doi: 10.1371/journal.pone.0284025 (PMC10079060; doi:10.1371/journal.pone.0284025)

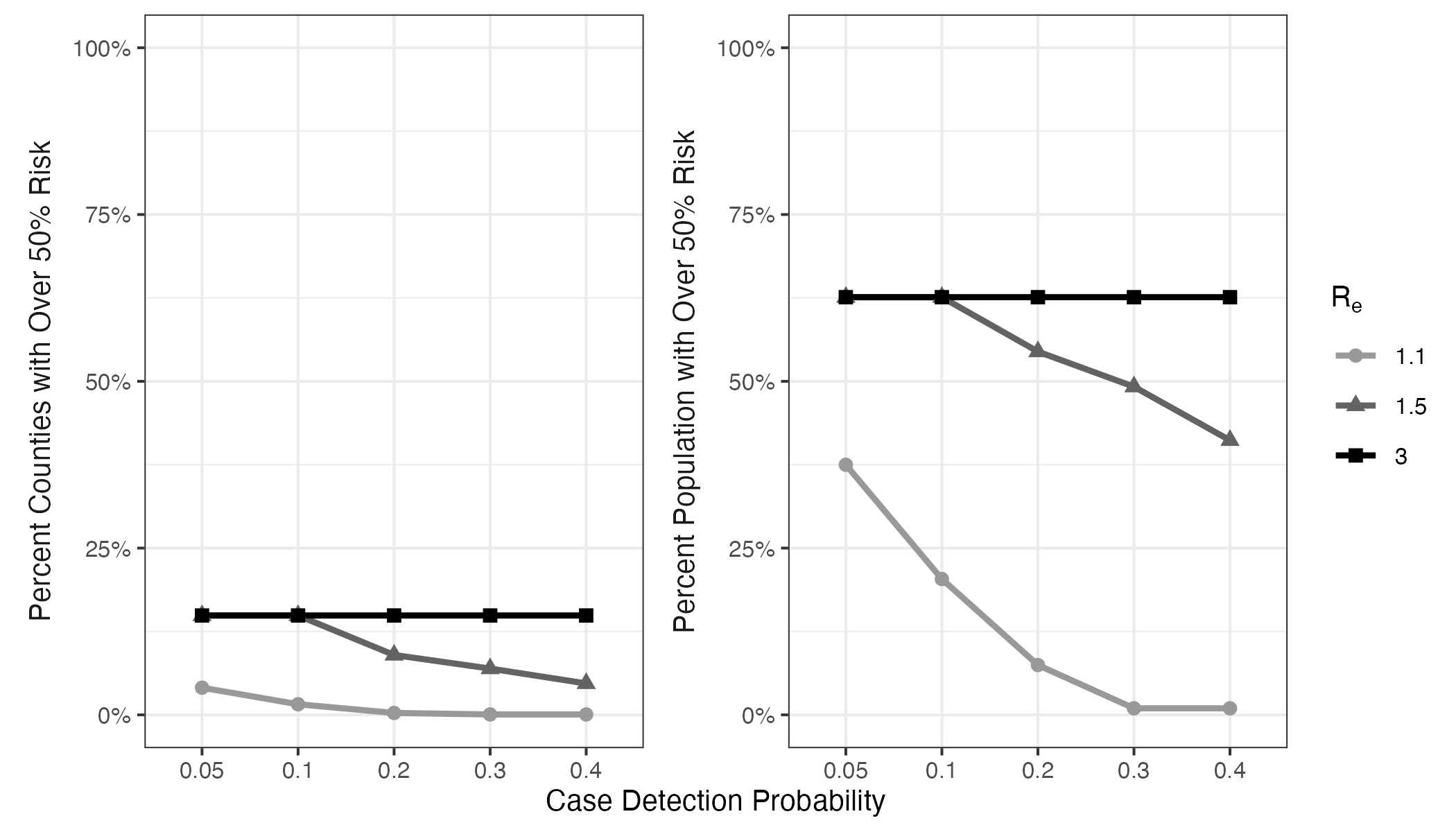

Supplement: S1 Fig — Percentage of US counties (left) or US population living in counties (right) that have greater than a 50% risk for sustained local transmission across varying assumed transmission rates (shade) and case detection probabilities (x-axis). (TIF) [file pone.0284025.s001.tif]

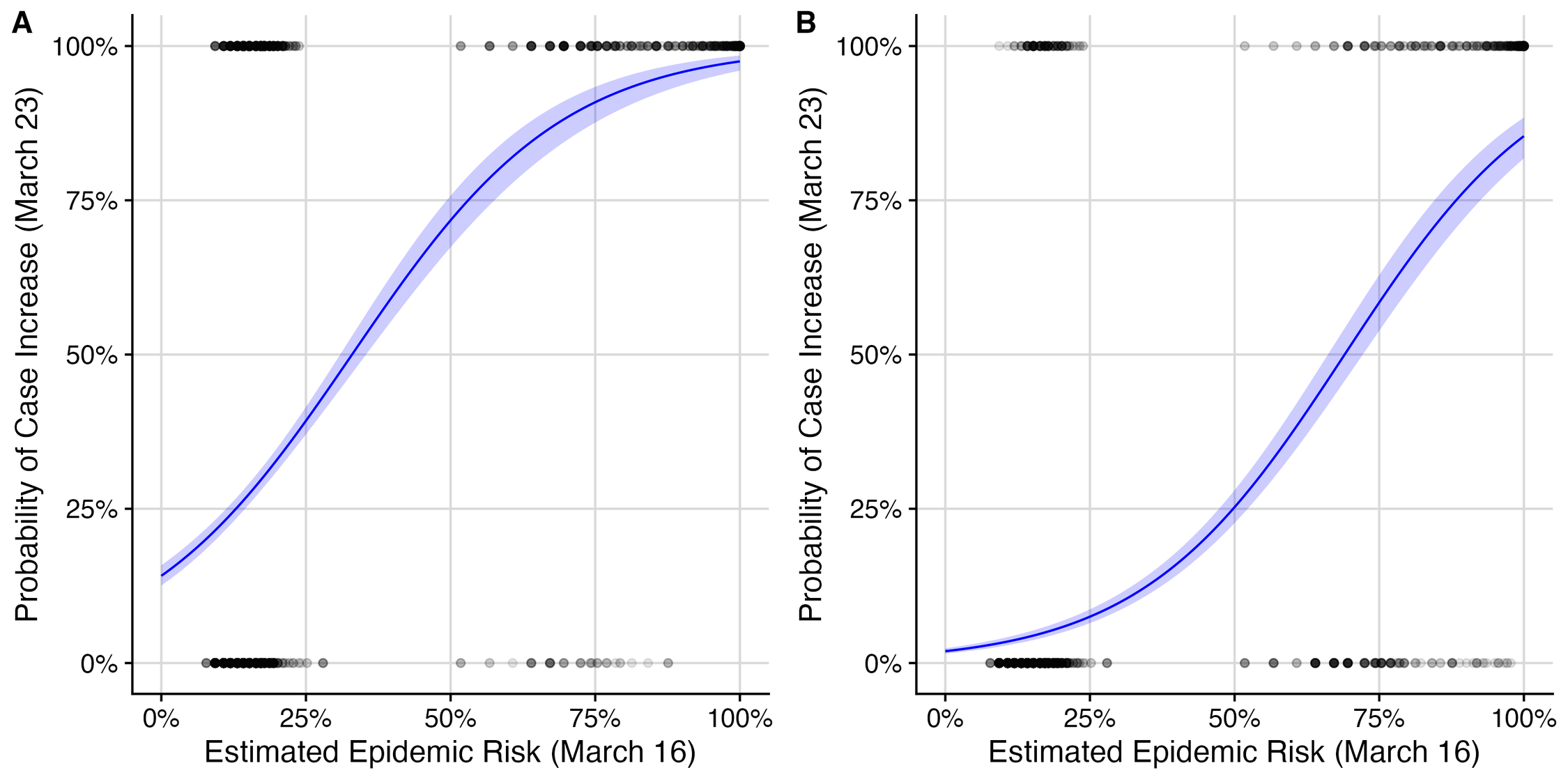

Supplement: S2 Fig — Points indicate the binary outcome for each county of whether it reported at least one (A) or five (B) new COVID-19 cases between March 16 and March 23. The bottom and top of the graph correspond to counties that did or did report such increases. The line and shading indicate the estimated mean (line) and 95% confidence interval (ribbon) resulting from a logistic regression relating actual one-week reported increase to estimated risk on March 16, 2020. We estimate that a 10% increase in model estimated epidemic risk for March 16 yields a 0.55 (95% CI 0.49–0.61) and 0.57 (95% CI 0.53–0.61) increase in the log odds that the county reported at least one or five additional cases in the following week, respectively. (TIF) [file pone.0284025.s002.tif]

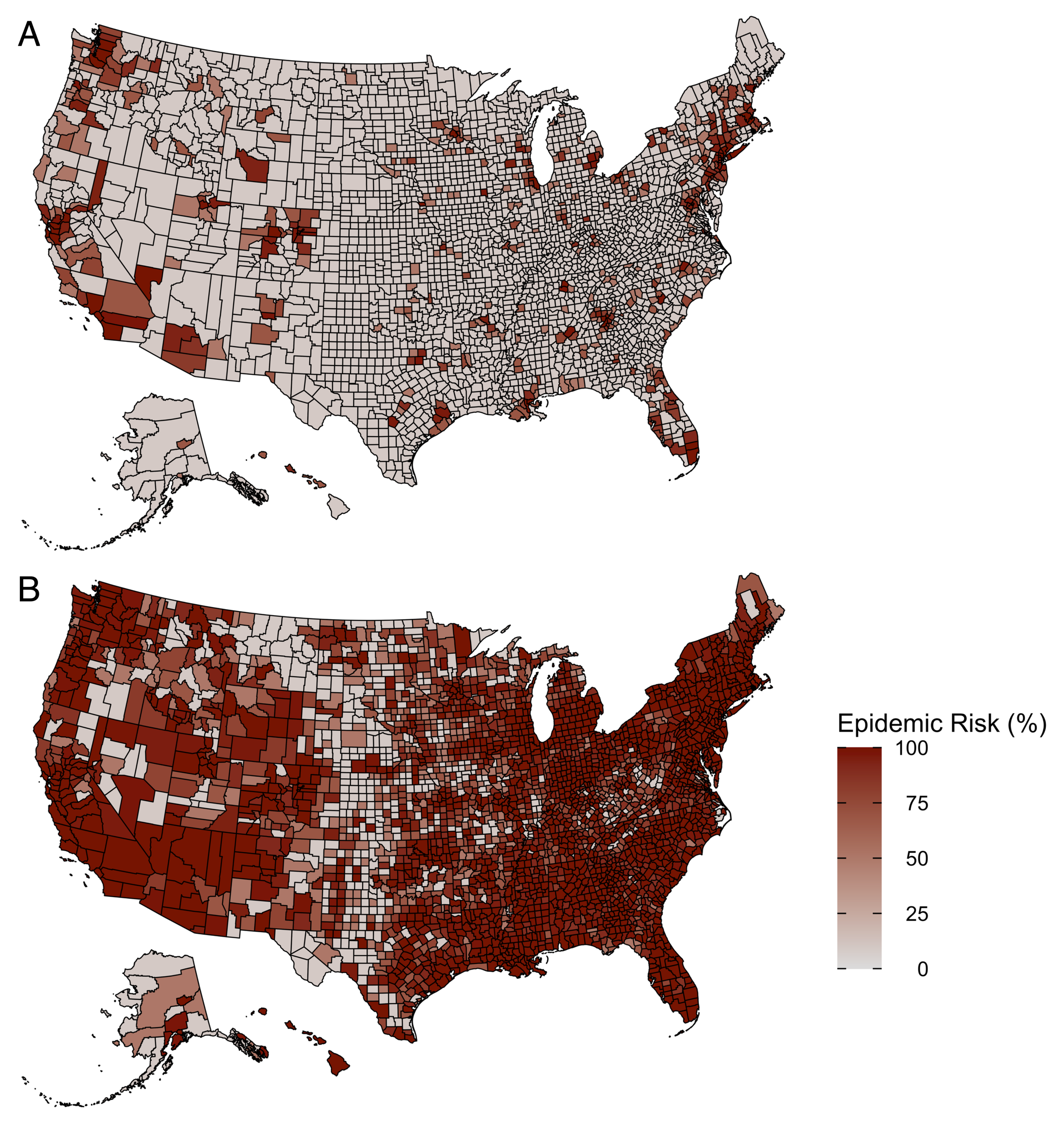

Supplement: S3 Fig — Original county-level estimates of ongoing COVID-19 epidemics assuming Re = 1.5 for (A) March 16, 2020 and (B) April 13, 2020. Estimated epidemic risk increased from 9% for zero cases to 50% when one case was detected and 100% for twenty-five or more cases. (A) By March 16, 2020, epidemic risk exceeded 50% in roughly 15% of the 3,142 counties covering 63% of the US population. (B) By April 13, 2020, we estimated that over 85% of US counties comprising 96% of the national population had at least a 50% chance of having an epidemic already underway. The estimates assume a 10% case detection rate and generation time of 6.0 days. (TIF) [file pone.0284025.s003.tif]

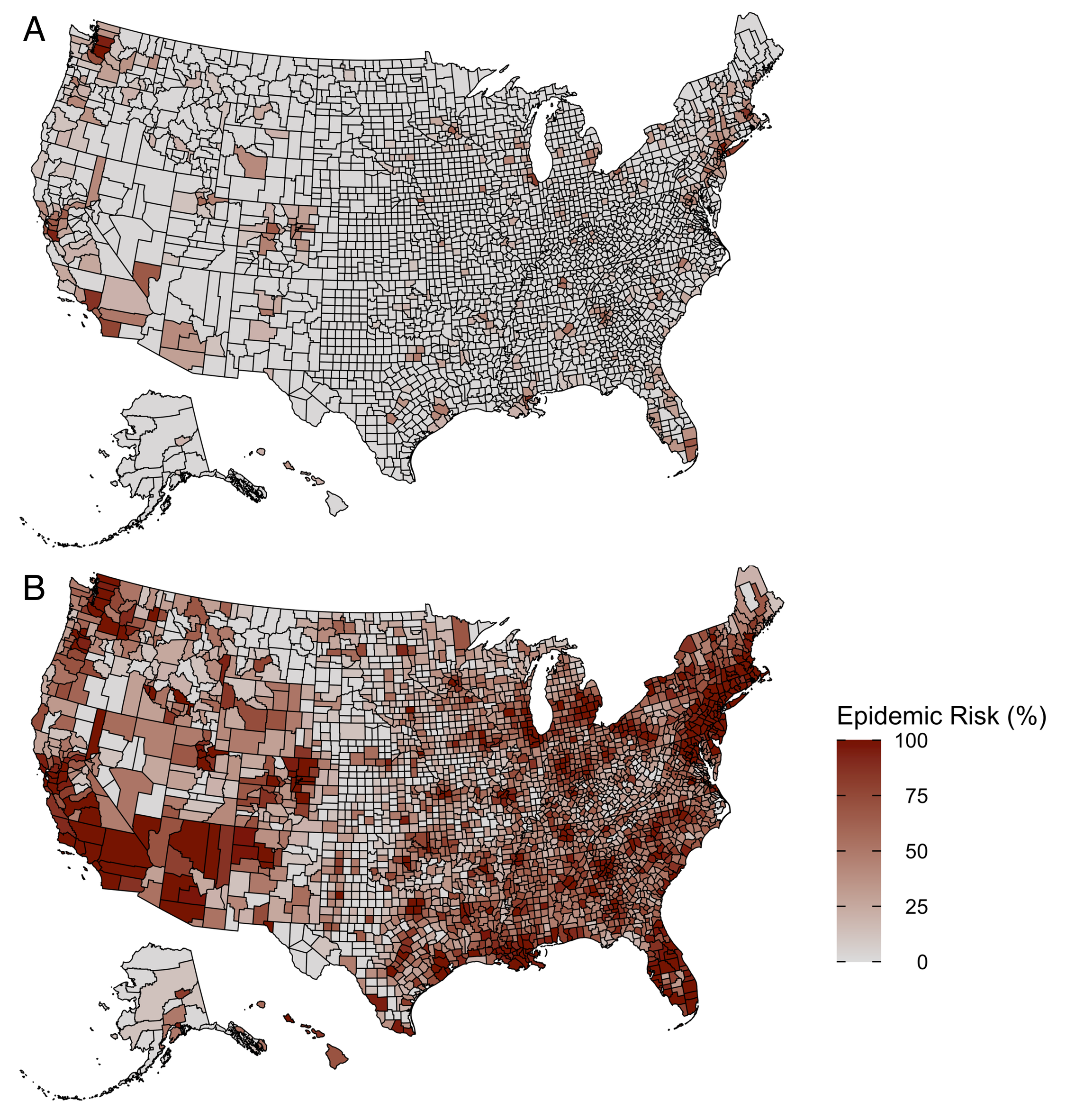

Supplement: S4 Fig — Original county-level estimates of ongoing COVID-19 epidemics assuming Re = 1.1 for (A) March 16, 2020 and (B) April 13, 2020. Epidemic risk increased from 2% for zero cases to 13% when one case was detected. An Re of 1.1 may be appropriate for counties with strict social distancing measures. The model assumes the original parameter estimates, including a 10% case detection rate and generation time of 6.0 days. (TIF) [file pone.0284025.s004.tif]

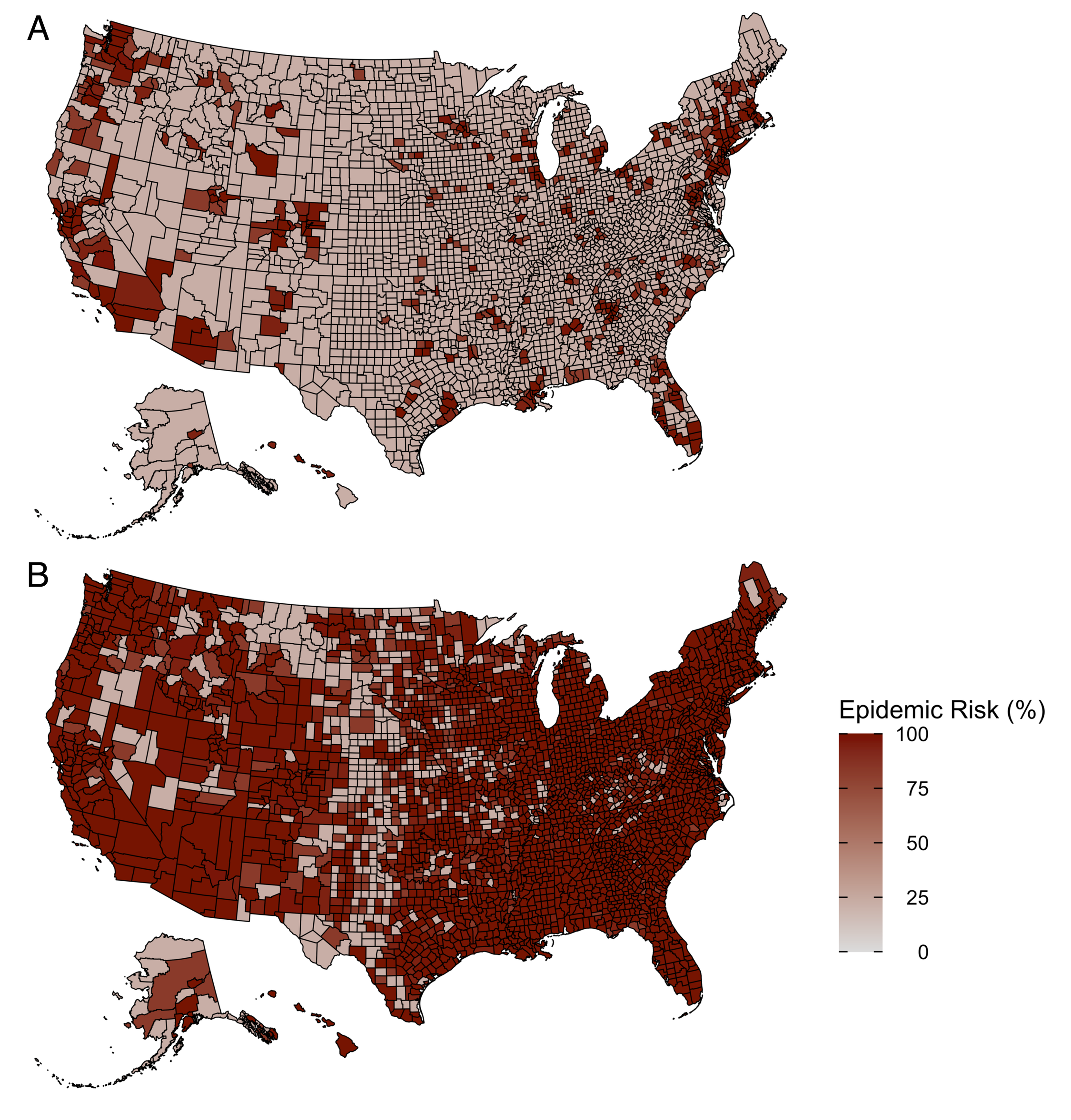

Supplement: S5 Fig — Original county-level estimates of ongoing COVID-19 epidemics assuming Re = 3.0 for (A) March 16, 2020 and (B) April 13, 2020. Epidemic risk increased from 22% for zero cases to 83% when one case was detected. The model also assumes the original parameter estimates, including a 10% case detection rate and generation time of 6.0 days. (TIF) [file pone.0284025.s005.tif]

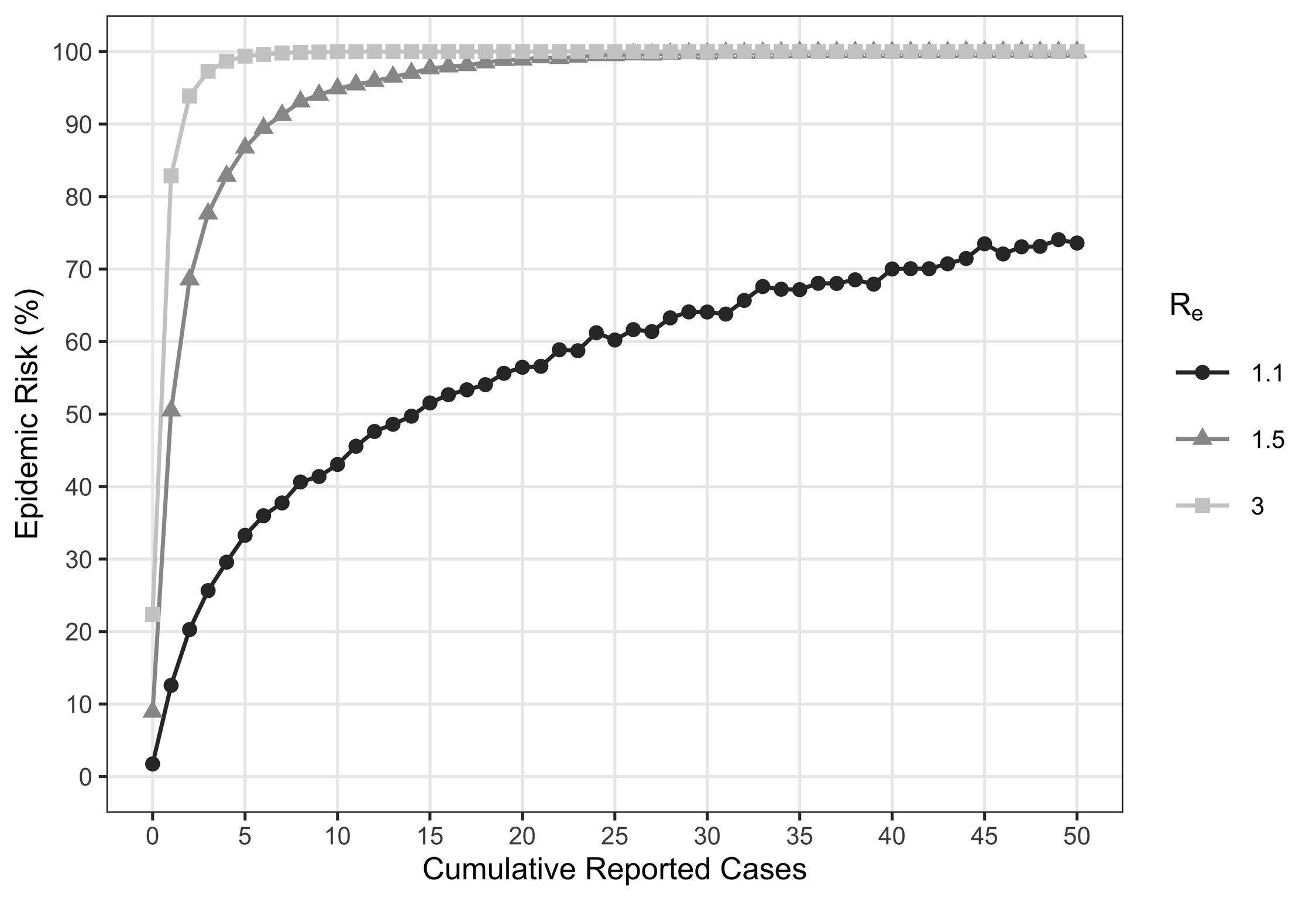

Supplement: S6 Fig — For a given number of reported cases, the estimated risk of an epidemic increased with Re. By the time a single case is reported, there is a 13%, 50% or 83% chance of an ongoing epidemic for an Re of 1.1, 1.5 or 3.0, respectively. (TIF) [file pone.0284025.s006.tif]

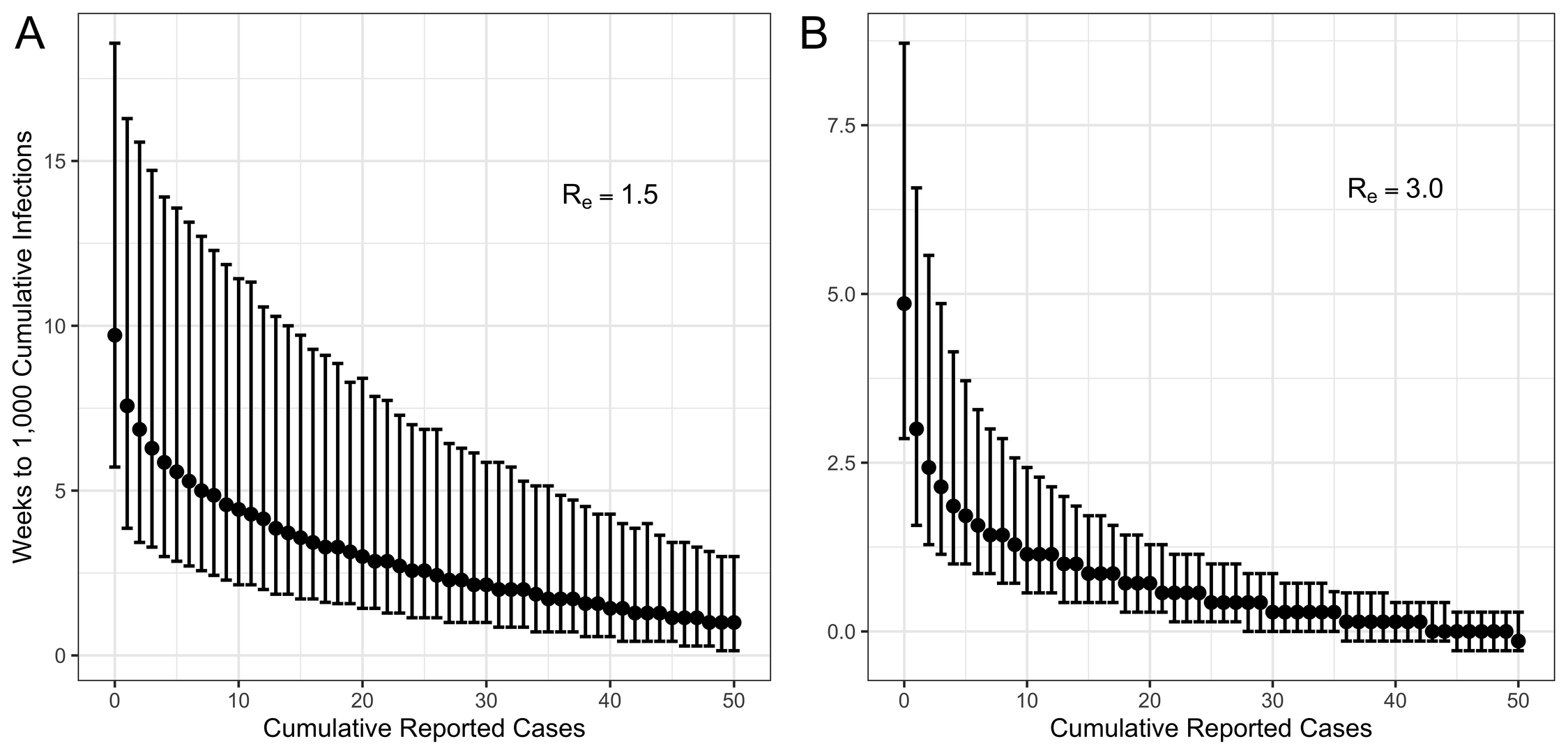

Supplement: S7 Fig — Expected time until epidemic exceeds 1,000 cumulative infections in a county, assuming (A) Re = 1.5 and (B) 3.0, a 10% case detection rate, and generation time of 6.0 days. For a given number of cumulative reported cases (x-axis), we estimate the median and 95% CI (error bars) number of weeks until the cumulative infections reach or exceed 1,000 for simulations classified as epidemics. (A) For Re = 1.5, when the first case is reported cumulative infections surpass 1,000 in 7.5 (95% CI 3.9–16.3) weeks; when the 10th case is reported, the expected lead time shrinks to 4.4 (95% CI 2.1–11.4) weeks. (B) Increasing Re to 3.0 there is a lag time of 3.0 (95% CI 1.6–6.6) weeks for the first case that decreases to 1.1 (95% CI 0.6–2.4) by the tenth case. Negative estimates suggest that 1,000 infections are reached prior to reporting a certain number of cumulative cases. The estimates are based on 100,000 stochastic simulations per Re. (TIF) [file pone.0284025.s007.tif]

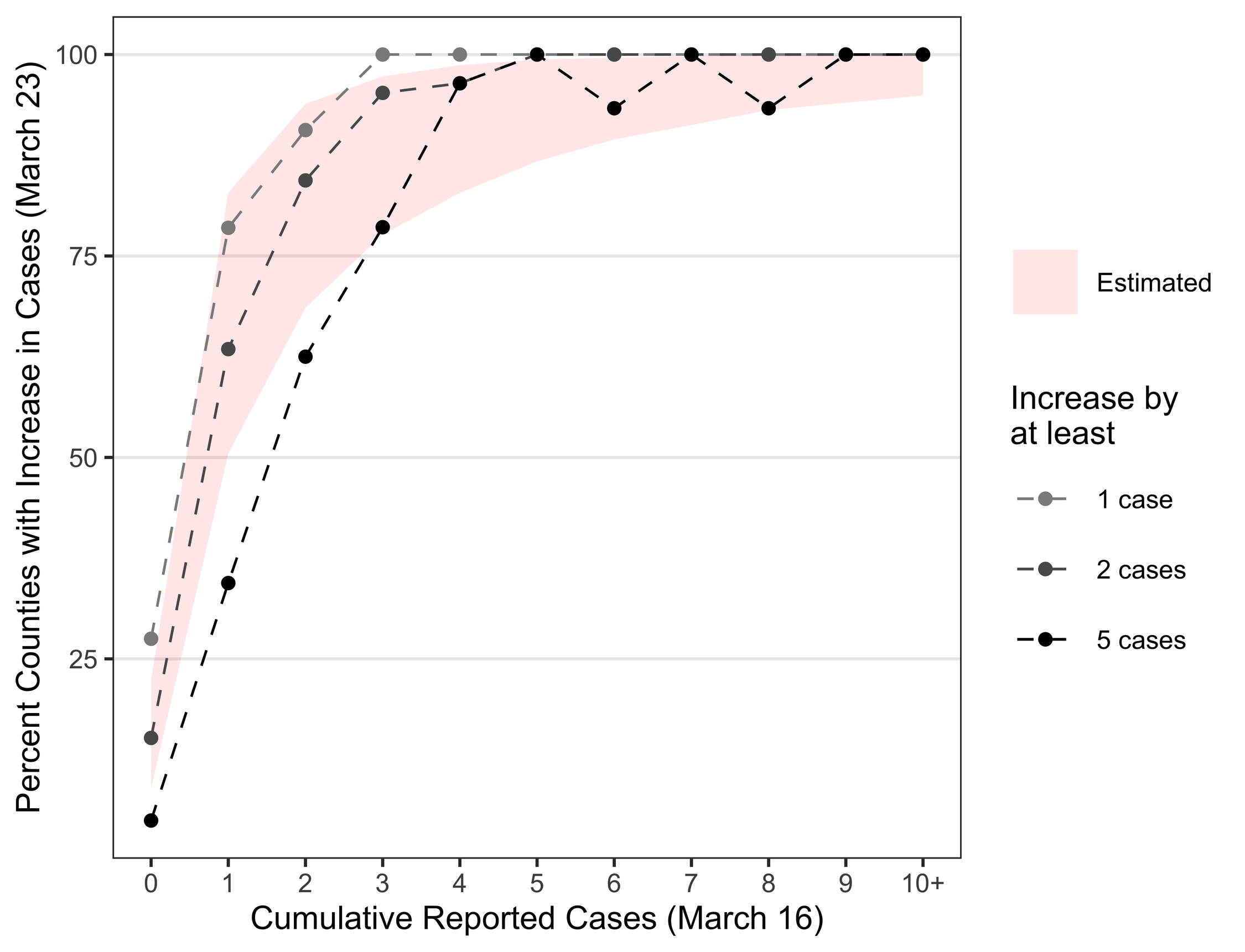

Supplement: S8 Fig — The light, medium and dark gray lines correspond to increases of at least one, two, or five new reported cases within one week, respectively. The red ribbon indicates the original model estimated epidemic risk, given the cumulative reported cases on March 16, 2020 indicated on the x-axis. The bottom and top of the ribbon correspond to estimates assuming Re = 1.5 and Re = 3.0, respectively. These estimates are calculated based on 100,000 simulations for each reproduction number, assuming a 10% case detection rate and a generation time of 6.0 days. The odds of a county detecting at least five new cases increased by 4.90 (95% CI 4.14–5.99) for every one unit increase in cases on March 16. For example, a county reporting only one case as of March 16 was roughly five times more likely to report at least six new cases a week later than a county with no previously reported cases. (TIF) [file pone.0284025.s008.tif]

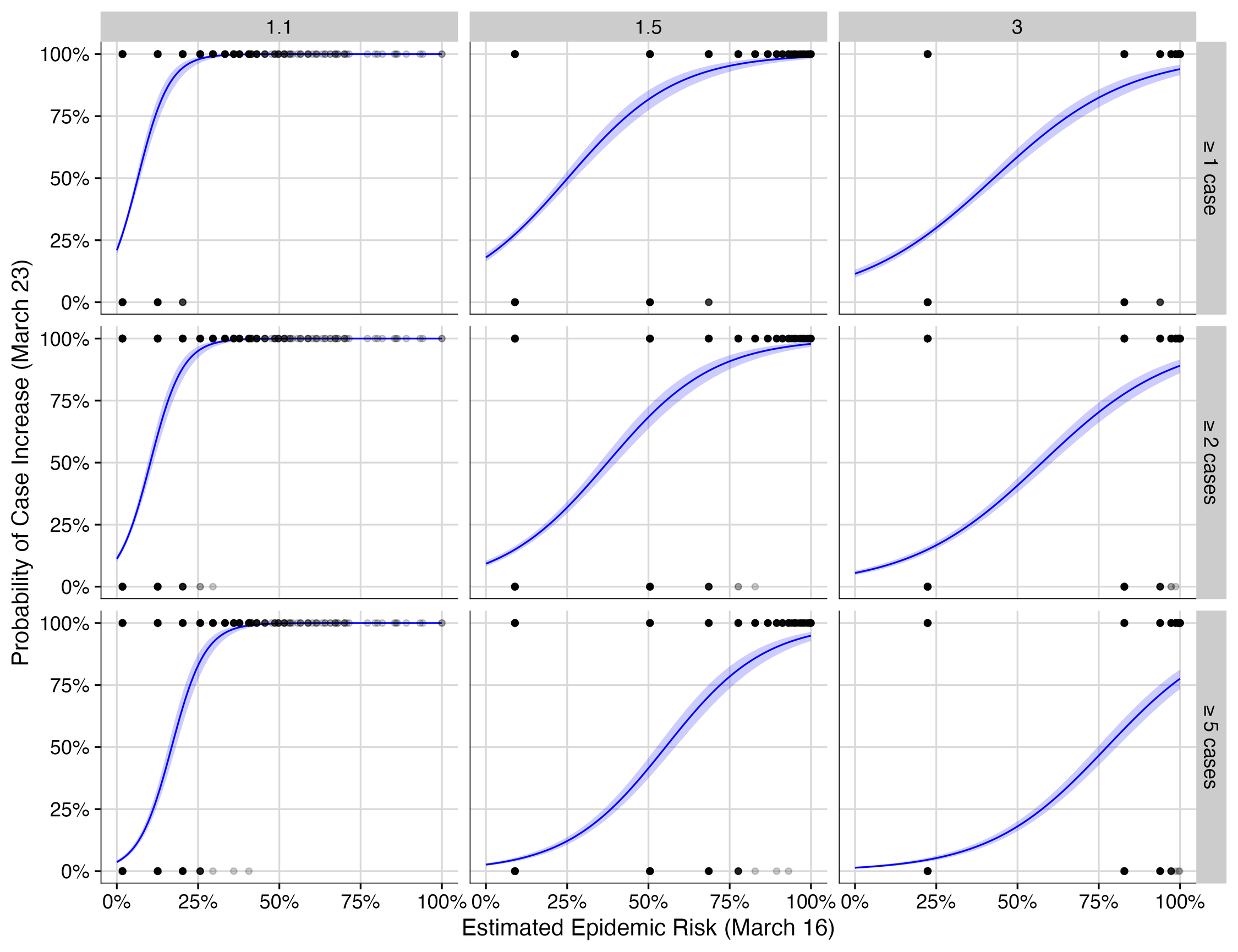

Supplement: S9 Fig — Points indicate the binary outcome for each county of whether it reported at least one, two, or five (rows) new COVID-19 cases between March 16 and March 23 under different assumed effective reproduction numbers (columns). Lines and ribbons indicate the estimated means and 95% confidence intervals for the fitted logistic regression models. (TIF) [file pone.0284025.s009.tif]

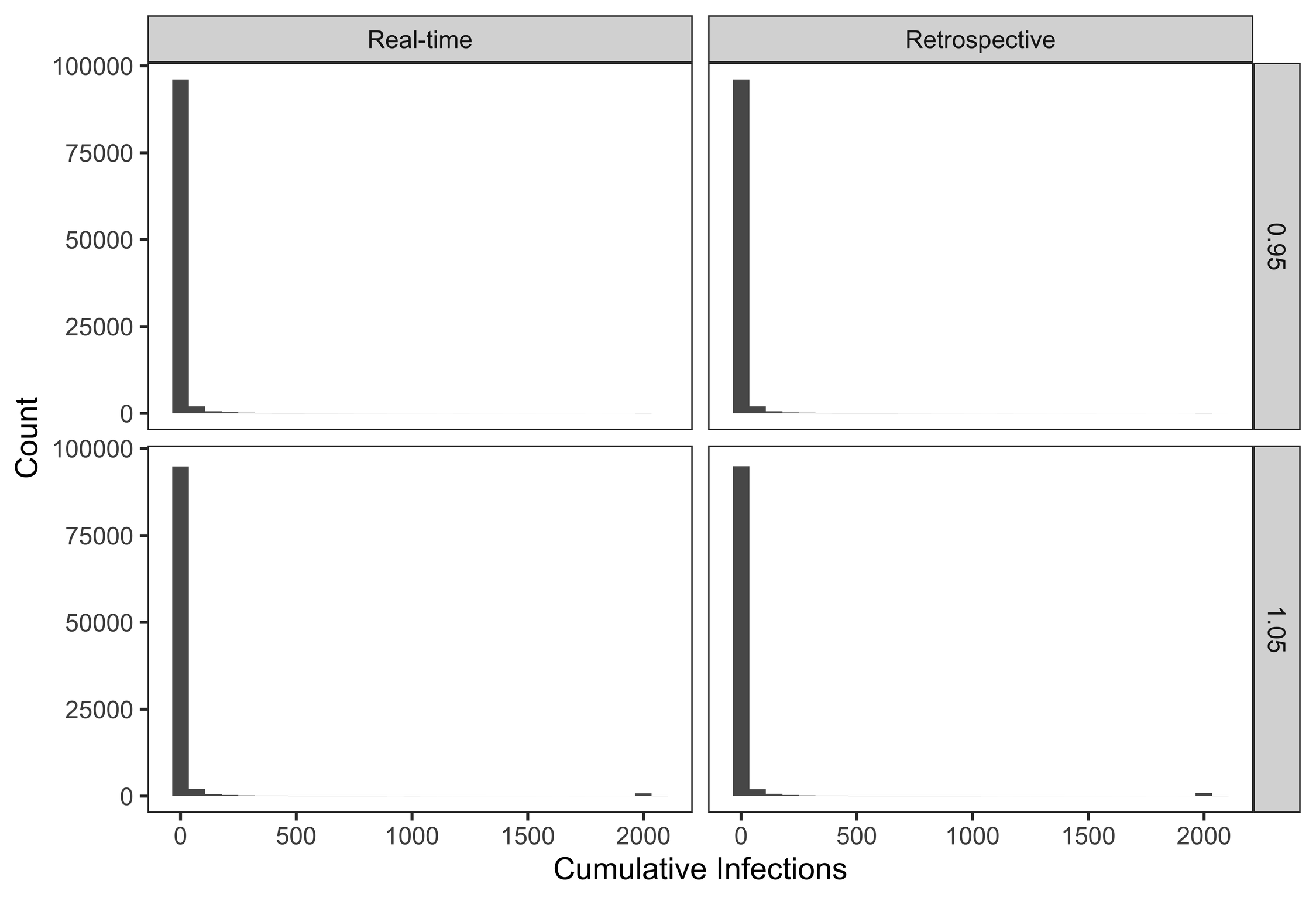

Supplement: S10 Fig — If simulations end too early, then more than expected can reach sufficient cumulative infections by random chance alone and not reflect true epidemics. By ending simulations at 2,000 cumulative infections we could confidently separate simulations with Re just below one (0.95, top row) from the epidemics of those with Re just above one (1.05, bottom row). For Re = 0.95 0.0% of original and 0.003% of retrospective simulations reached 2,000 cumulative infections and met a minimum prevalence of 50 new infections. As Re increased to 1.05, 0.11% of original and 0.10% of retrospective simulations were classified as epidemics. Ending simulations at 2,000 cumulative infections and requiring a minimum prevalence of 50 new infections per day is sufficient to distinguish self-limiting simulations from self-sustaining. (TIF) [file pone.0284025.s010.tif]
